# Modified natural kaolin clay as an active, selective, and stable catalyst for methanol dehydration to dimethyl ether

**DOI:** 10.1038/s41598-022-13349-0

**Published:** 2022-06-07

**Authors:** Mohamed Abd El-Aal, Abd El-Aziz Ahmed Said, Mohamed H. Abdallah, Mohamed Nady Goda

**Affiliations:** 1 Catalysis and Surface Chemistry Lab, Chemistry Department, Faculty of Science, Assiut University, Assiut, 71516 Egypt; 2 Chemistry Department, Faculty of Science, Al-Azhar University, Assiut, 71524 Egypt

**Keywords:** Catalysis, Energy

## Abstract

In this work, the production of dimethyl ether (DME) from methanol over natural kaolin clay modified through impregnation with various percentages of H_2_SO_4_, WO_3_, or ZrO_2_ catalysts was investigated. The prepared catalysts were characterized via X-ray fluorescence, X-ray diffraction, Fourier transform infrared spectroscopy, scanning electron microscopy, and N_2_-sorption analysis. The acidity of these catalysts was determined through the dehydration of isopropyl alcohol and the chemisorption of pyridine. The catalytic activity performance revealed that the addition of modifiers into kaolin enhanced the latter’s activity toward DME production. In addition, the kaolin clay modified with 10 wt% ZrO_2_ exhibited excellent activity of 98% conversion with 100% selectivity at 275 °C. Moreover, this catalyst could proceed the reaction for a long time (6 days) without any noticeable deactivation. The remarkable improvement in the catalytic performance achievement was well correlated with the acidity and the structure of the catalysts.

## Introduction

Environmental pollution and the looming depletion of oil reserves have driven intense research to produce alternative clean energy sources such as H_2_ and dimethyl ether (DME). DME is one of the most promising candidates for replacing petroleum oil because of its excellent environmental behavior, with energy density of 31.7 MJ/kg, and low auto-ignition temperature^1^. DME can also be used as an efficient intermediate for various industrially important chemicals^2^, aerosol propellant in the cosmetic industry^3^, coolant, and source of H_2_ for fuel cells^4^. However, there are several drawbacks to adopting DME as a diesel alternative^1^. When compared to diesel fuel, DME has a lower viscosity, which can lead to leaks and component damage. DME has a low boiling point, which necessitates the use of a pressured system to keep the fuel in a liquid form. Furthermore, DME has an energy density of 31.7 MJ/kg, compared to roughly 45 MJ/kg for diesel, therefore despite higher energy efficiency, DME still requires a bit more fuel injected per cycle. As a result, altering the fuel tank and the fuel distribution system is one of the most difficult aspects of converting diesel vehicles to DME^2^. Moreover, the cost of DME production from biomass equals 3 times higher than that of diesel fuel, while from natural gas equals 90–135% of the diesel fuel cost.

DME can be produced in two ways: directly from syngas (CO, CO_2_, and H_2_) and indirectly from methanol. The catalytic dehydration of methanol vapor to DME is the promising and more efficient route, considering thermodynamics and economy^5^. Regardless of the synthesis route, a solid acid catalyst is required to dehydrate methanol to DME. To date, numerous acid catalysts have been used, e.g., ɣ-alumina^6–8^, modified ɣ-alumina^9–11^, η-alumina^12^, zeolite^13–15^, modified zeolite^16^, sulfated zirconia^17^, and aluminum phosphate^18,19^. The most used materials are ɣ-alumina and zeolite because of their high performance in terms of methanol conversion and DME selectivity. Despite this advantage, ɣ-alumina and zeolites are deactivated by water^20^ and coke formation^21^, respectively. The deactivation of ɣ-alumina by water can be reduced by increasing the hydrophobicity of the support^22^. Therefore, searching for alternative solid acid catalysts with high water resistance (hydrophobic materials), minimal carbonaceous formation, and cost-effective^23–25^ is essential.

Clay catalysts are eco-friendly, abundant, cheap, and reusable^26^. Kaolin is a clay material with various industrial applications, such as in catalysis, photocatalysis^27^, decolorization^28^, adsorption^29^, ceramics, paper coating, plastic fillers, paint extenders, and cement^30^. Kaolinite is the main clay mineral present in kaolin, which reportedly contains a two-layered structure where a sheet of octahedrally coordinated AlO_2_(OH)_4_ is connected to a tetrahedral coordinated SiO_4_ sheet^31^. Kaolinite cannot be directly used as a catalyst because of its limitations, such as impurities, porosity, low surface area, and acidity^32^. The open literature gives various methods for improving the catalytic activity of kaolinite. The most important modification methods are chemical activation and mechanochemical and thermal treatments^32^. Modified kaolinite reportedly possesses an acidity suitable for the dehydration of methanol to DME^33,34^. In this issue, Solyman et al.^33^ modified kaolinite via intercalation with alumina, which was then chemically modified by H_2_O_2_ and mechanochemically modified by ball milling with and without CaSO_4_. They found that the kaolinite sample modified by ball milling in the presence of CaSO_4_ showed the highest activity with an 84% DME yield at 400 °C using gas hourly space velocity **(**GHSV) of 8 ml/g/h. The catalytic performance of kaolinite modified by urea was also studied^34^. The kaolinite complex treated by boiling in water showed the best activity with an 87% DME yield at 400 °C using GHSV of 8 ml/g/h. The maximum DME yields in the previously mentioned studies are obtained at high temperatures. However, high temperatures are unfavorable owing to the exothermic nature of the reaction and the thermodynamic equilibrium limited conversion^35^. Thus, modifying kaolin to be an active, selective, and stable catalyst in the dehydration of methanol to DME at relatively low temperatures is of great interest.

In this paper, two series of catalysts were prepared and used for methanol dehydration. The first was obtained by treating kaolinite with H_2_SO_4_ at different percentages. The second was obtained by intercalating kaolinite with WO_3_ and ZrO_2_ at different percentages. The modified samples were characterized via X-ray diffraction (XRD), Fourier transform infrared (FTIR), surface area, scanning electron microscopy (SEM), and acidity. The relationships between the catalytic performance of the modified samples with their acidity and physicochemical properties were well correlated.

## Results and discussion

### Catalyst characterization

**Figure 1:**
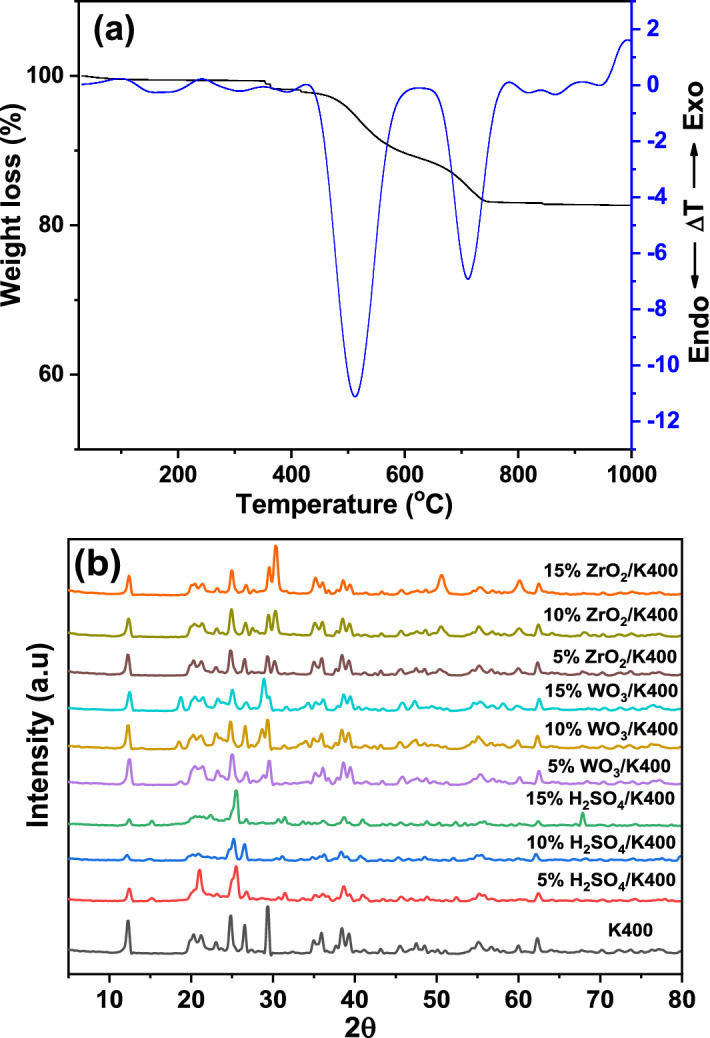
(**a**) TG and DTA curves of raw Kaolin and (**b**) XRD diffraction patterns of kaolin and modified kaolin catalysts calcined at 400 °C.

The thermal behavior of the raw kaolin in the temperature range of 25–1000 °C was checked by TG and DTA techniques and the results are shown in Fig. 1a. Examination of the TG curve revealed that, three weight losses were observed. The first weight loss (1.5%) lies in the temperature range of 25 to 350 °C and accompanied by two broad endothermic peaks on the DTA curve, minimized at 175 and 305 °C. This weight loss is attributed to the evolution of physically adsorbed water from the surface of the kaolin sample. The second and third weight losses (11 and 6%) were occurred in the temperature ranges of 400–650 °C and 650–750 °C and were accompanied by endothermic peaks on the DTA curve located at 518 and 713 °C, respectively. These events are ascribed to the dehydration of kaolin to form metakaolin according to the following equation^36^:

$$ {\text{2Al}}_{{2}} {\text{Si}}_{{2}} {\text{O}}_{{5}} \cdot \left( {{\text{OH}}} \right)_{{4}} \to {\text{2Al}}_{{2}} {\text{Si}}_{{2}} {\text{O}}_{{7}} + {\text{ 4H}}_{{2}} {\text{O}}. $$

On increasing the heating temperature up to 1000 °C, an exothermic peak on the DTA curve was observed at 980 °C. This exothermic peak can be attributed to the conversion of the amorphous metakaolin into a crystalline phase (Si_3_Al_4_O_12_) spinel according to the following equation^36^:

$$ {\text{2 Al}}_{{2}} {\text{Si}}_{{2}} {\text{O}}_{{7}} \to {\text{Si}}_{{3}} {\text{Al}}_{{4}} {\text{O}}_{{{12}}} + {\text{ SiO}}_{{2}} . $$

The structural changes in the kaolin material due to impregnation with different percentages of H_2_SO_4_, WO_3_, and ZrO_2_ were studied using the XRD technique. The XRD profiles of the K400 sample and of those treated with H_2_SO_4_, WO_3_, and ZrO_2_ are shown in Fig. 1b. The K400 sample shows well-defined reflections at 2*θ* values of 12.3°, 20.3°, 23.1°, 24.9°, 38.3° and 39.3° which well-matched with the data bank of kaolinite as a major phase (COD card No: 9009230). Diffraction peaks of quartz (COD card No: 1011176) could be found at 2*θ* = 50.2° and 60.1°. Illite (COD card No: 9013732) was detected at 2*θ* = 26.6°. Muscovite (COD card No: 1000042) was also observed at 2*θ* = 35°. In addition, Calcite (COD card No: 1010928) was detected at 2θ of 29.5°, 43.2°, 47.6° and 48.6°. The XRD diffraction patterns of the kaolin modified by 5–15 wt% of H_2_SO_4_ showed that the peak intensity of kaolin clay gradually decreased with increasing H_2_SO_4_ percentage. This behavior indicates that acid treatment leads to structural disorders, which affects the crystallinity of the kaolin^37^. In addition, the XRD peak that assigned to calcite phase at 2*θ* = 28.9° is completely disappeared due to the reaction occurred between CaCO_3_ and sulfuric acid. The intercalation of WO_3_ also slightly lowered the intensities of the characteristic’s peaks of the kaolin clay. Moreover, new reflections appeared at 2*θ* = 23.3°, 23.8°, 38.1° and 38.9°, which typically characterize triclinic WO_3_ (COD card No: 1010618). The intensities of these reflections gradually increased with increasing WO_3_ loading on the kaolin. On the other hand, the XRD patterns of the kaolin samples incorporated with different loading percentages of ZrO_2_ were similar to that of K400, indicating that the intercalation with ZrO_2_ did not damage the kaolin crystal structure. This means that all the diffraction peaks of kaolin still appeared after the intercalation; nevertheless, the peak intensities slightly decreased. This result reflects that ZrO_2_ was successfully incorporated into the kaolin clay support. New peaks came out at 2*θ* = 30.2°, 50.7°, and 60.2°, and their intensities increased with increasing ZrO_2_ weight percentage were observed. These new peaks corresponded to the tetragonal phase of ZrO_2_ (COD card No: 1525706), which may have a role in enhancing the catalytic activity of these samples.

**Figure 2:**
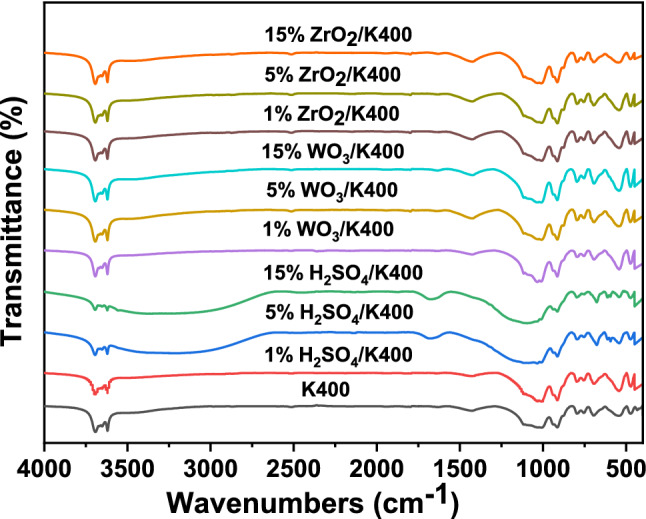
FTIR spectra of kaolin and modified kaolin catalysts calcined at 400 °C.

The FTIR spectra of the unmodified K400 and of the modified kaolin calcined at 400 °C (Fig. 2) were taken to analyze the vibrational bands and the interface interaction potentially responsible for promoting catalytic activity. The K400 sample showed all the characteristic vibration bands of kaolinite. The bands at 3694, 3670, and 3652 cm^−1^ were assigned to an inner-surface OH-stretching modes of kaolinite^38^, whereas the band at 3620 cm^−1^ was associated with the stretching mode of the inner hydroxyl group of kaolinite^39^. The small and broad band at 3450 cm^−1^ may be attributed to water physisorbed on the surface of kaolin^40^. The band at 1430 cm^−1^ was ascribed to a vibrational mode of CO_3_^2−^ group which is due to the presence of calcite^41^. The bands at 1632, and 915 cm^−1^ were attributed to water functional group (H–O–H)^41^ and the O–H deformation of Al–O–H inner surface hydroxyl group^42,43^, respectively. The band around 1010 cm^−1^ corresponded to ν(Si–O stretching mode)^44^, whereas the band appeared at 694 cm^−1^ was assigned to (Si–O out-of-plane bending)^45^. Meanwhile, the bands assigned at 796, 755, 695 and 544 cm^−1^ were ascribed to the lattice vibrations^39^. The bands at 470 and 430 cm^−1^ were attributed to the vibration of Si–O (in-plane) bending associated with OH^46^ and the Si–O bending vibration^32^, respectively. After the kaolin was modified by low ratios of H_2_SO_4_ (1–3 wt%), a little variation in the band patterns was observed. However, with a further increase in the acid loading (5–15 wt%), some bands appeared, disappeared, or shifted, and their intensities also changed. In this issue, the structural hydroxyl vibrations bands are progressively decreased due to the dehydroxylation process which caused by the penetration of acid protons into the kaolin layers and attack the structural OH group^47^. Moreover, an increase in the wavenumber from 1632 cm^−1^ in the K400 sample to 1667 cm^−1^ in the modified kaolin^48^, and the latter band was more intense. Furthermore, the stretching vibration of –OH from water adsorbed was appeared broader, more intense and at much lower wavenumber 3100 cm^−1^^49^. These results confirmed that the modification of kaolin by H_2_SO_4_ influences the OH bending and stretching vibrations of water. The change in the position and the intensities of the latter two bands in the modified kaolin may have a role in the catalytic dehydration of methanol to DME. Meanwhile, two doublet bands also appeared at 610 and 594 cm^−1^, which may be due to the bending modes of SO_4_^−2^^50,51^. The asymmetric and symmetric stretching modes of O=S=O also detected at 1120–1230 cm^−1^ and 1010–1080 cm^−1^, respectively, and these bands are overlapped with that of the Si–O–Si band^52^. The enhancement in the band’s intensities in 960–1545 cm^−1^ region with increasing the loading of H_2_SO_4_ on kaolin was observed. This enhancement may be due to the increase of the concentrations of the stretching vibration of the S=O bond, the symmetric vibrations of Si–O–S bridges^53^, and the increase in the amorphous silica percentages^54^. It is also noted that the band corresponded to the presence of calcite (1430 cm^−1^) disappeared due to the reaction of CaCO_3_ with H_2_SO_4_. This observation agrees with that obtained from the XRD peak analysis; the diffraction peaks of calcite disappeared in the H_2_SO_4_ modified kaolin samples. The FTIR spectra of the kaolin treated with different percentages of WO_3_ are presented in Fig. 2. No apparent change in the intensities and the positions of the characteristic bands of kaolin was observed. This may be due to the weak intensity of the characteristic bands of WO_3_ or the band corresponding to W–O–W that appeared at a position of 800 cm^−1^ which like that of the quartz band. Figure 2 shows negligible change in the intensities or the positions of the characteristics bands of kaolin when it was impregnated with ZrO_2_. No characteristic bands of ZrO_2_ appeared in these samples because of the very low intensities of the bands related to ZrO_2_ itself or the bands corresponding to Zr–O–Zr or Zr–O–Si have positions similar to those of the band’s characteristics of the K400 structure. Accordingly, the absence of the FTIR bands due to the presence of WO_3_ and ZrO_2_ in the modified samples indicates that both oxides are highly dispersed or incorporated into the kaolin framework.

**Figure 3:**
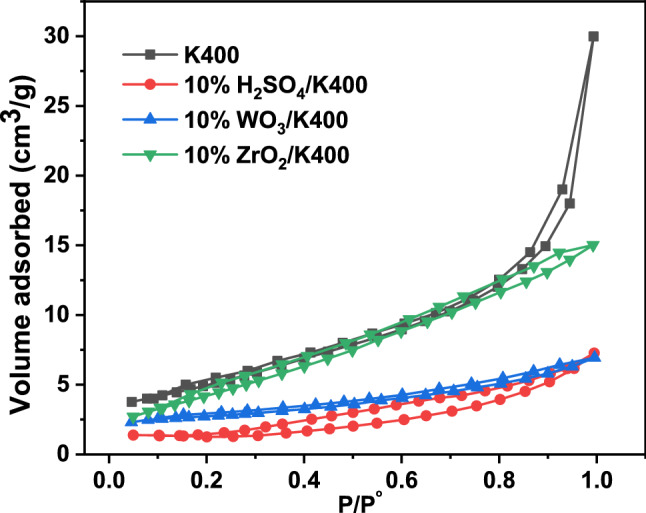
N_2_ adsorption–desorption isotherms of kaolin and modified kaolin catalysts calcined at 400 °C.

BET measurements were conducted to determine the effects of the H_2_SO_4_, WO_3_, and ZrO_2_ additions on the texture properties of the K400 sample. The isotherms of the K400 and the modified kaolin clay catalysts (Fig. 3) belonged to typical type II isotherms and possessed an H3 hysteresis loop. According to IUPAC classification, this demonstrates that the K400 and modified K400 catalysts have micropores formed owing to the presence of aggregates of plate-like particles, giving rise to slit-shaped pores^55^. Table 1 shows the *S*_BET_ and porosity characteristics of the samples. The calculated value of the *S*_BET_ of the K400 clay is 25.2 m^2^/g. Upon the impregnation of the K400 sample with H_2_SO_4_, the specific surface area and the pore volume fell regularly because of the partial collapse of the clay structure due to the strong acid penetration of the crystal structure^56^. The modification of the K400 sample with WO_3_ also reduced the pore volume and specific surface area. These findings indicate that the WO_3_ blocked the pores of the K400 sample, which are responsible for such decrease. Conversely, the modification of the K400 sample with 1–3 wt% ZrO_2_ decreased the specific surface area and pore volume. The *S*_BET_ value increased upon a further increase in the ZrO_2_ content but was still lower than that of the K400 sample. This increase in the specific surface area may be attributed to the formation of tetragonal phases of zirconia on the surface of the kaolin clay, as confirmed previously in the XRD section.

**Table 1:** Texture properties of kaolin and modified kaolin catalysts calcined at 400 °C.

Catalyst	Isotherm type	S_BET_ (m^2^/g)	Total pore volume (cc/g) × 10^–2^	Pore radius (Å)
K400	II	25.2	5.1	21.1
1% H_2_SO_4_/K	II	5.6	1.8	20.8
3% H_2_SO_4_/K	II	3.8	1.7	20.7
5% H_2_SO_4_/K	II	3.6	1.7	20.6
10% H_2_SO_4_/K	II	3.6	1.5	20.5
15% H_2_SO_4_/K	II	2.5	1.5	20.5
1% WO_3_/K	II	9.9	3.1	20.9
3% WO_3_/K	II	5.7	3.8	20.8
5% WO_3_/K	II	7.6	3.1	20.9
10% WO_3_/K	II	7.2	2.4	20.9
15% WO_3_/K	II	4.7	1.9	20.6
1% ZrO_2_/K	II	6.7	1.9	20.9
3% ZrO_2_/K	II	5.5	1.8	20.8
5% ZrO_2_/K	II	12.2	3.2	20.7
10% ZrO_2_/K	II	20.3	4.0	20.7
15% ZrO_2_/K	II	18.7	3.2	15.6

**Figure 4:**
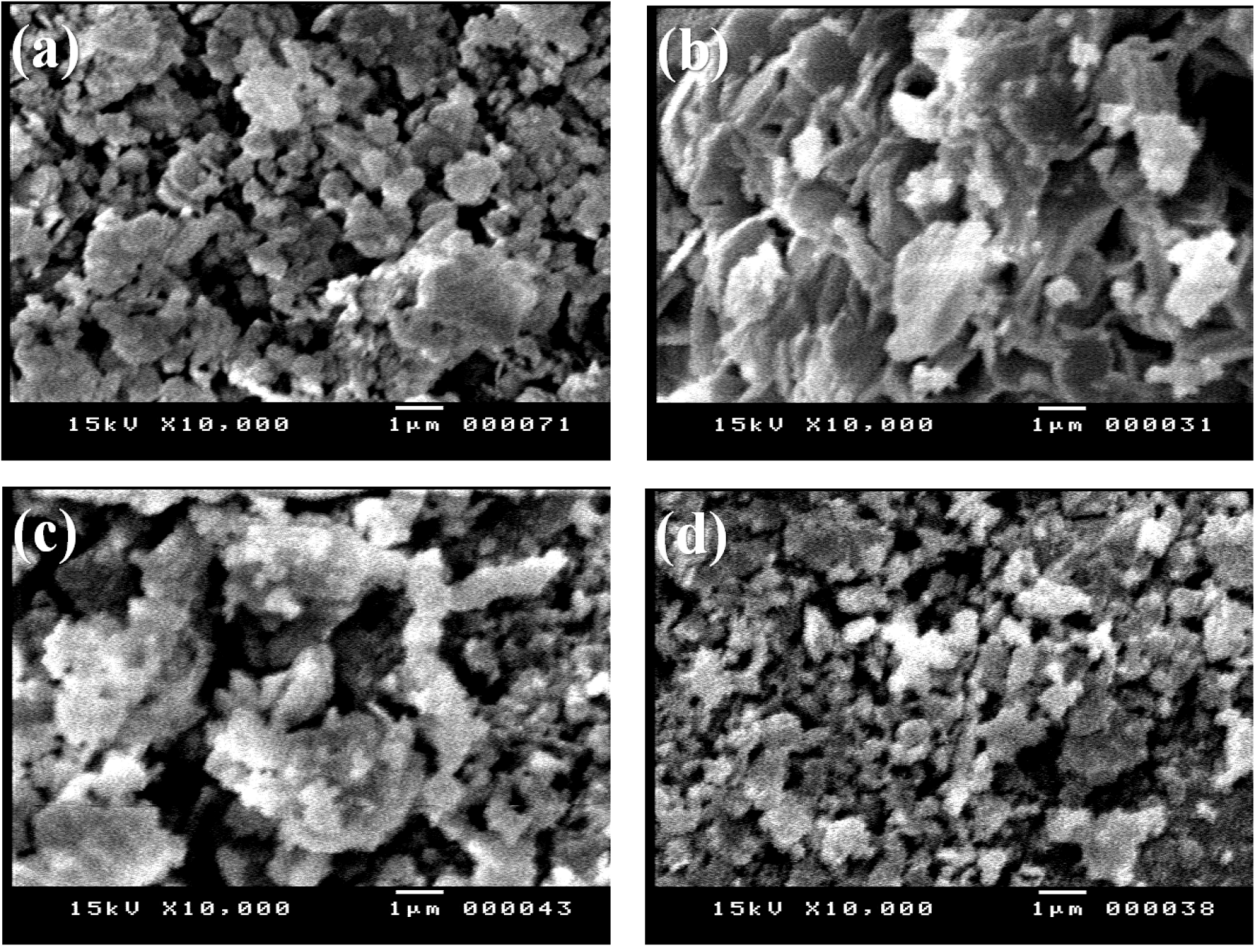
SEM images of (**a**) K400, (**b**) 10% H_2_SO_4_/K400, (**c**) 10% WO_3_/K400, and (**d**) 10% ZrO_2_/K400 catalysts calcined at 400 °C.

The morphologies of the K400, 10% H_2_SO_4_/K400, 10% WO_3_/K400, and 10% ZrO_2_/K400 catalysts were studied using SEM, and the obtained images are shown in Fig. 4. The K400 sample has aggregates of semispherical structure and agglomerated particles. The SEM image of 10% H_2_SO_4_/K400 shows flaky particles stacked together. Meanwhile, 10% WO_3_/K400 has large, agglomerated particles. The SEM image of 10% ZrO_2_/K400 is like that of the K400 sample, but with a slightly smaller particle size.

### Acidity measurement

#### IPA dehydration

**Figure 5:**
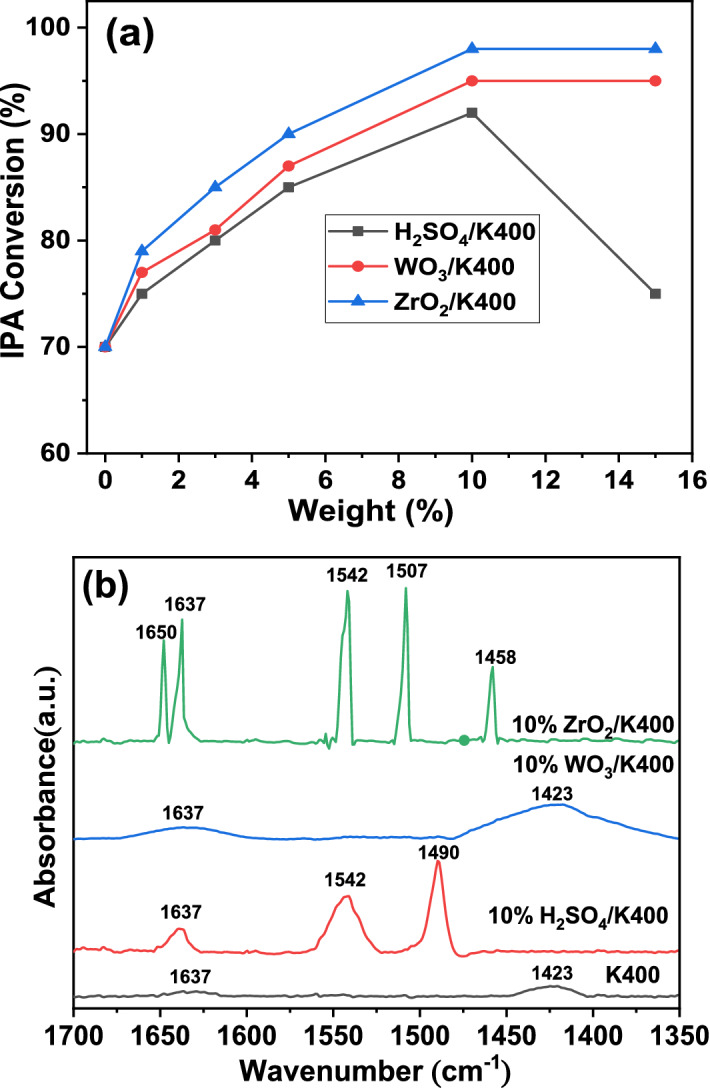
Catalytic dehydration of IPA over (**a**) kaolin and modified kaolin catalysts, and (**b**) FTIR spectra of pyridine adsorption on kaolin and modified kaolin catalysts calcined at 400 °C. Error bar = 2%.

The activity of the catalyst toward the dehydration of IPA has been reported to be a good measure of the catalyst acidity^10,11,57^. Hence, the acidity of the kaolin and modified kaolin was explored by dehydrating IPA at 200 °C and the results are shown in Fig. 5a. Under our working conditions, all the catalysts have good IPA dehydration activity with 100% selectivity to propene. By closely checking the results, we found that the K400 catalyst exhibited an IPA conversion of ≈ 70%. After kaolin was impregnated with different percentages of H_2_SO_4_, the catalytic dehydration of IPA improved to ≈ 92% over 10% H_2_SO_4_/K400. Upon increasing the H_2_SO_4_ content to 15 wt%, an observable decrease in the IPA conversion to ≈ 75% was detected. The decrease in the activity of the catalysts in the presence of high H_2_SO_4_ ratios may be related to the breakdown of the crystalline structure of the kaolin catalyst, as observed from the XRD patterns. The impregnation of kaolin with different percentages of ZrO_2_ improved the catalytic dehydration of IPA to a maximum conversion of ≈ 98% observed over the 10% ZrO_4_/K400 catalyst. The WO_3_-modified kaolin catalysts showed the same behavior as that of the ZrO_2_-modified kaolin, with a maximum conversion of ≈ 95% observed over the 10% WO_3_/K400 catalyst. We concluded from these results that the acidity of the modified kaolin is higher than that of the unmodified kaolin.

#### Pyridine-FTIR adsorption (Py-FTIR)

The Brønsted (B) and Lewis (L) acid sites of the most active catalysts of the modified kaolin in the IPA dehydration compared with that of the unmodified (K400) were identified using the Py-FTIR technique and the results are illustrated in Fig. 5b. The FTIR signals located at 1423, 1458, and 1490 cm^−1^ corresponded to pyridine coordinated at (L) sites. The signals that arose at 1507, 1542, 1637, and 1650 cm^−1^ were related to pyridine molecules protonated at (B) sites^58,59^. The FTIR spectrum of the K400 sample pre-saturated with pyridine exhibited two signals centered at 1423 and 1637 cm^−1^, which can be attributed to the (L) and (B) acid sites bound to pyridine, respectively. Wahyuni et al.^60^ found that the surface of kaolin had both (L) and (B) acid sites. The (L) acid site on 10% H_2_SO_4_/K400 was revealed by the presence of a signal at 1490 cm^−1^, whereas the (B) acid sites were identified by the appearance of signals at 1542 and 1639 cm^−1^. The 10% WO_3_/K400 catalyst had both (L) acid sites (1423 cm^−1^) and (B) acid sites (1637 cm^−1^). Similarly, the 10% ZrO_2_/K400 catalyst had (L) acid sites (1458 cm^−1^) and (B) acid sites (1507, 1542, 1637, and 1650 cm^−1^). In conclusion, all the four tested catalysts had both (L) and (B) acid sites in different amounts. Additionally, the modification of kaolin with 10% of H_2_SO_4_, WO_3_, or ZrO_2_ enhanced the total acidity by more than fourfold (Table 2). The increase in the total acidity of 10% H_2_SO_4_/K400 is due to the substitution of exchangeable cations of Na^+^ and Mg^2+^ by H^+^ ions and the formation of bridging hydroxyl groups between nearest neighbor Al atoms of the Si atom^61^. Conversely, the increase in the total acidity of 10% WO_3_/K400 and 10% ZrO_2_/K400 is due to the presence of triclinic WO_3_ and tetragonal ZrO_2_ in the modified kaolin, respectively, as confirmed previously from the XRD results. Finally, based on the total acidity, the samples take the following order: K400 < 10% H_2_SO_4_/K400 < 10% WO_3_/K400 < 10% ZrO_2_/K400.

**Table 2:** Distribution of Lewis (L) and Brønsted acid (B) sites and the total acidity on K400, 10% H_2_SO_4_/K400, 10% WO_3_/K400, and 10% ZrO_2_/K400 catalysts calcined at 400 °C.

Catalyst	L%	B%	Total acidity (mmol/g)
K400	61.5	38.5	0.02
10% H_2_SO_4_/K400	36.5	63.5	0.80
10% WO_3_/K400	84.2	15.8	0.90
10% ZrO_2_/K400	11.7	88.3	1.20

### Catalytic dehydration of methanol

**Figure 6:**
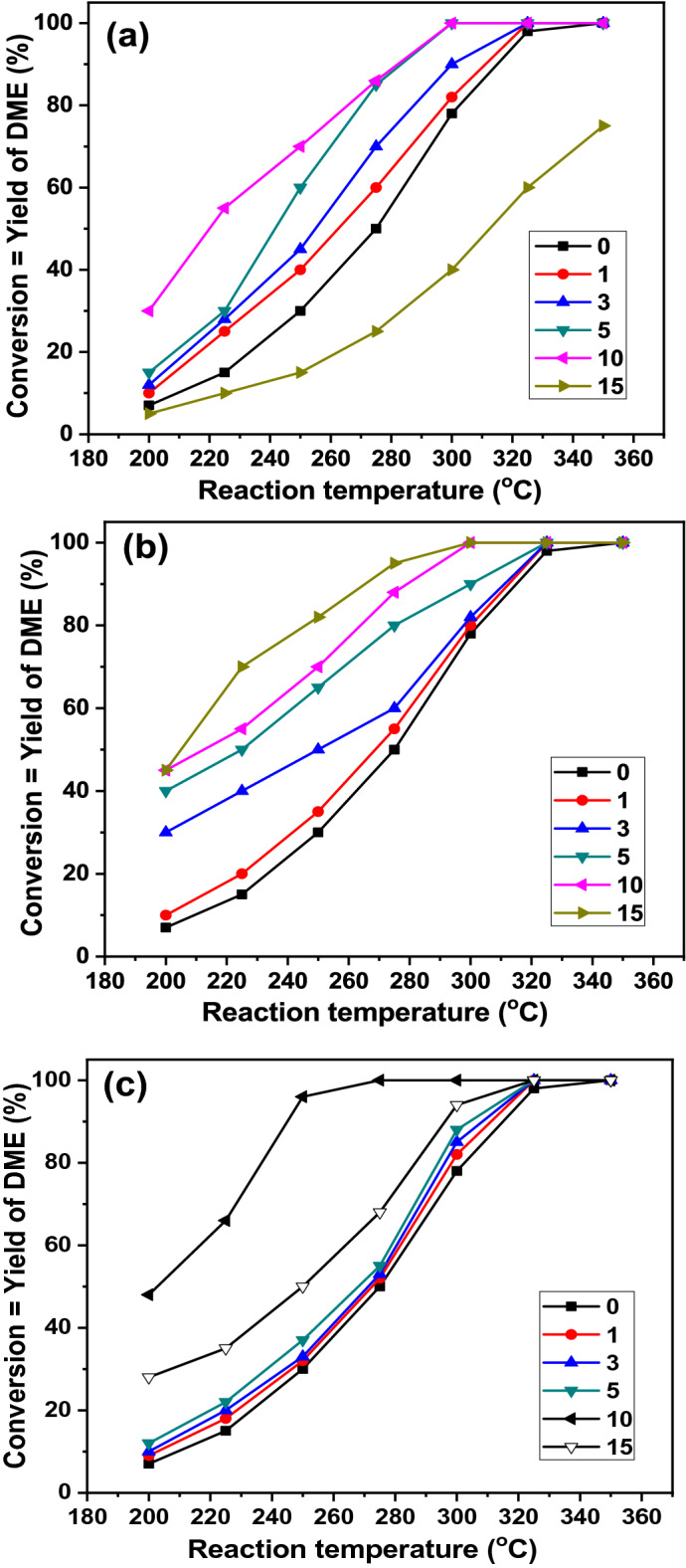
Catalytic dehydration of methanol into DME over kaolin and modified kaolin with different percentages of (**a**) H_2_SO_4_, (**b**) WO_3_, and (**c**) ZrO_2_ calcined at 400 °C, at different reaction temperatures. Error bar = 2%.

The catalytic dehydration of methanol over the K400 and modified kaolin catalysts was conducted to measure the catalytic activity in terms of methanol conversion in the temperature range of 200–350 °C (Fig. 6). As seen in Fig. 6, all catalysts exhibited nearly similar behaviors, where the methanol conversion increased with increasing reaction temperatures from 200 to 350 °C with 100% selectivity to DME. The observed differences in catalytic activity could be related to the acidity, the hydrophobicity^22^, and the texture properties of the catalysts. The K400 sample is an active catalyst with a maximum methanol conversion and DME yield ≈ 98% obtained at 350 °C. However, it shows very low activity at 200 °C (only 7% methanol conversion). The kaolin clay was impregnated with different percentages of H_2_SO_4_ to improve its catalytic activity at relatively low temperatures, and the results are shown in Fig. 6a. It shows that at 200 °C, acid impregnation significantly increases the catalytic activity from 7 to 30% over the kaolin modified by 10 wt% of H_2_SO_4_. However, when the acid percentage was further increased to 15 wt%, a sharp decrease in the catalytic activity was observed. Moreover, the conversion of methanol increased linearly by increasing the percentages of H_2_SO_4_ to 10 wt%. However, the maximum methanol conversion with DME yield ≈ 98% was obtained over the catalysts modified with 5 and 10 wt% of H_2_SO_4_ at a reaction temperature of 300 °C. At 300 °C, the methanol conversion rates for this series of catalysts follow the sequence 15% H_2_SO_4_/K400 < K400 < 1% H_2_SO_4_/K400 < 3% H_2_SO_4_/K400 < 5% H_2_SO_4_/K400 = 10% H_2_SO_4_/K400. Conversely, at 200 °C, the methanol conversion increased to 45% over the 10% WO_3_/K400 catalyst (Fig. 6b). The maximum methanol conversion ≈ 98% and DME yield ≈ 98% were achieved over the 10% WO_3_/K400 catalyst at 300 °C. Moreover, at this temperature, the activities of the catalysts follow the order K400 < 1% WO_3_/K400 < 3% WO_3_/K400 = 5% WO_3_/K400 < 10% WO_3_/K400 = 15% WO_3_/K400. Additionally, in the case of the ZrO_2_-modified kaolin series of catalysts (Fig. 6c), at 200 °C, the methanol conversion increased from 7 to 48% with the increased percentage loading of ZrO_2_ to 10%. At 300 °C, the catalytic activity follows the order K400 < 1% ZrO_4_/K400 < 3% ZrO_2_/K400 < 5% ZrO_2_/K400 < 15% ZrO_2_/K400 < 10% ZrO_2_/K400. However, the maximum methanol conversion is nearly 98% with a 98% yield of DME was obtained over the 10% ZrO_4_/K400 catalyst at 275 °C.

**Figure 7:**
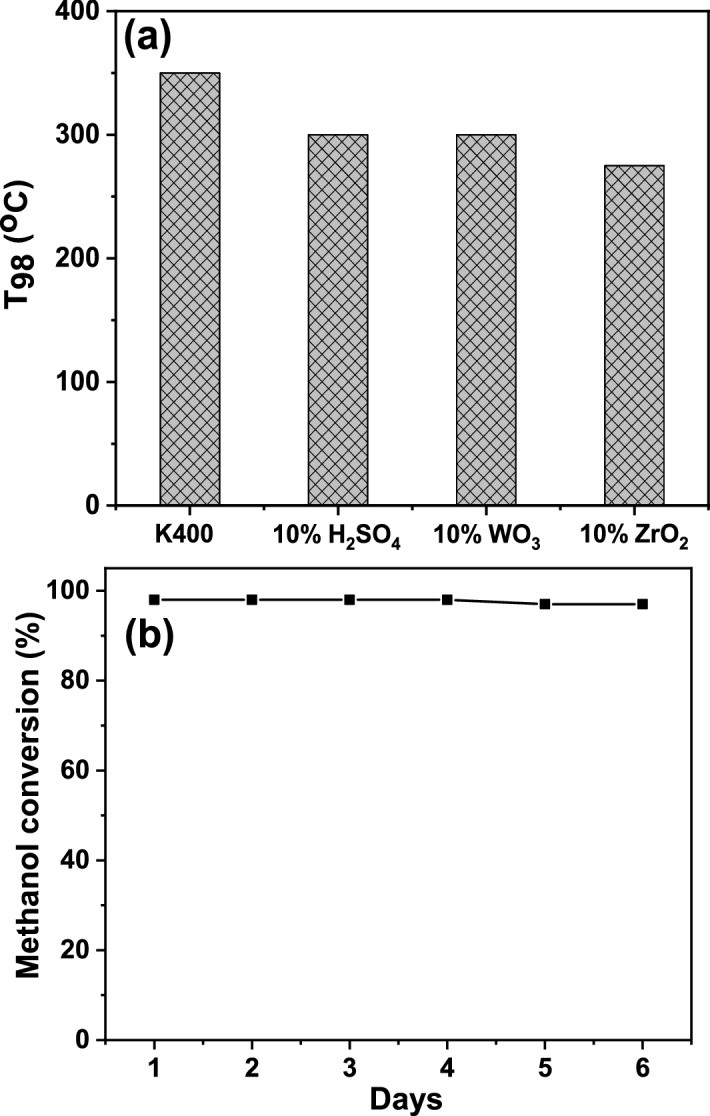
(**a**) T_100_ values of K400, 10% H_2_SO_4_/K400, 10% WO_3_/K400, and 10% ZrO_2_/K400 catalysts calcined at 400 °C and, (**b**) long-term stability of 10% ZrO_2_/K400 catalyst calcined at 400 °C at a reaction temperature of 275 °C.

A plot of the temperature where the complete dehydration of methanol to DME (T_98_) for the K400 and modified kaolin catalysts is presented in Fig. 7a_,_ to compare the catalytic performance of the most active catalysts. The reaction temperature required to obtain the complete conversion of methanol to DME takes the following order: K400 > 10% H_2_SO_4_/K400 = 10% WO_3_/K400 > 10% ZrO_2_/K400. Therefore, the 10% ZrO_2_/K400 catalyst had the highest catalytic activity at relatively lower temperature (275 °C). This high catalytic activity may be attributed to the formation of tetragonal zirconia which accompanied by high acidity values, as mentioned previously in the XRD and acidity sections. Our group previously found that the formation of the tetragonal phase of zirconia caused the greatest enhancement in the acidity and the catalytic activity of sulfated zirconia^17^. A similar enhancement was also obtained by modifying FePO_4_ with 10% of ZrO_2_^62^. Kou et al.^63^ found that Zr-pillared clay samples are quite effective in dehydrating methanol to DME and hydrocarbons because of their porosity and acidity. Methanol dehydration to DME was studied on Zr-loaded P-containing mesoporous activated carbon catalysts^64^. The catalyst with 5.25% Zr-loading showed the highest methanol conversion ≈ 69% with 95% selectivity to DME. The high catalytic activity of this catalyst may be attributed to the increased acidity due to the formation of zirconium phosphate active species. Chmielarz et al.^65^ found that the intercalation of porous clay heterostructures with Zr increased the conversion of methanol to 73% with nearly 100% selectivity to DME at 325 °C. They assumed that the acid sites on this catalyst are in the clay mineral layers and are related to the incorporated Zr^4+^ cations.

### Catalyst stability

Catalyst stability in terms of conversion and selectivity is the most important parameter reflecting catalyst quality. Hence, the stability of the most active catalyst (10% ZrO_2_/K400) for methanol dehydration to DME was tested for 6 days at 275 °C. The results are depicted in Fig. 7b. The maximum conversion of methanol (98%) to DME with 100% selectivity was observed to be nearly unchanged for a long duration. This means that the catalyst did not exhibit any sign of deactivation under the reaction conditions, which was confirmed by completely matching the XRD patterns of the fresh and the spent catalyst (Fig. S1). Thus, this catalyst has excellent stability to produce DME from methanol.

Table 3 shows the comparison of the catalytic performance of the modified kaolin catalysts with that of other clay minerals. Despite the different reaction conditions used in this study and other studies, the modified kaolin presented herein showed a remarkable activity and selectivity than those of the clay catalysts described in the literature. Additionally, the modified kaolin with ZrO_2_ achieved the complete conversion of methanol with nearly 100% selectivity to DME at relatively low temperature (≈ 275 °C). Moreover, this result is in comparable with the other catalysts published in the literature. This advantage makes modified kaolin with H_2_SO_4_, WO_3_ and ZrO_2_ are promising catalysts for dehydrating methanol to DME.

**Table 3:** Comparison of the activity of the most active catalysts with that published in the literature.

Catalyst	Reaction temperature (°C)	GHSV (ml/g/h)	Methanol conversion (%)	Selectivity to DME (%)	References
CaSO_4_ modified kaolinite	400	8.000	84.6	84	^33^
Urea kaolinite complex	400	8.000	87.5	87	^34^
Co/kaolinite	300	7200	81.7	< 80	^66^
Allophane	300	12,000	83.0	97	^67^
Porous clay doped with Al	325	12.000	80	100	^65^
Red mud	300	–	68	100	^68^
K10 montmorillonite	300	–	80	100	^69^
Cu doped Zr-PILCs	240	3000	44.5	95.2	^63^
10% H_2_SO_4_/K400	300	6000	98	100	This work
10% WO_3_/K400	300	6000	98	100	This work
10% ZrO_2_/K400	275	6000	98	100	This work

## Conclusion

Natural kaolin clay impregnated with different percentages of H_2_SO_4_, WO_3_, or ZrO_2_ exhibited high catalytic performance in the gas-phase dehydration of methanol to DME at relatively low temperatures. The results revealed that the kaolin structure and acidic properties were greatly influenced by the ratios of the modifiers used. The observed differences in the catalytic activity of the modified and unmodified kaolin could be attributed to the acidity, hydrophobicity, and the texture properties of the catalysts. The catalyst containing 10 wt% of ZrO_2_ exhibited excellent activity of 98% conversion with 100% DME selectivity. The high catalytic performance of this catalyst was correlated to the formation of tetragonal zirconia, which accompanied with high acidity values. Additionally, this catalyst displayed long-term stability toward methanol dehydration to DME up to 6 days without deactivation. Thus, it is a potentially suitable catalyst for producing DME from methanol at a temperature of 275 °C. The obtained results in this study will open doors for the preparation of highly active, eco-friendly, and cost-effective catalysts based on natural kaolin and their application in acid-catalyzed reactions.

## Materials and methods

### Materials

H_2_SO_4_ (95%), isopropyl alcohol (IPA) (99.98%), pyridine (99.3%), methanol, phosphotungstic acid, H_3_O_40_PW_12_·*x*H_2_O, and zirconyl nitrate, ZrO(NO_3_)_2_·2H_2_O were provided by Sigma-Aldrich, Germany, and were used as received without any further purification.

### Synthesis of modified kaolin

The kaolin clay used in this study was collected from the Aswan area, Egypt, and sieved to an average particle size of 0.25 mm. The chemical analysis of the raw sample through X-ray fluorescence (Table 4) shows that the sample had a high percentage of SiO_2_ and Al_2_O_3_, with a SiO_2_/Al_2_O_3_ ratio equal to 1.5. The sample was thermally activated in a muffle furnace at 400 °C for 3 h to remove the physically adsorbed water and improve its surface properties before use. It was then labeled as K400. The modified kaolin was prepared by impregnating the K400 sample with an aqueous solution of known quantities of H_2_SO_4_ or phosphotungstic acid or zirconyl nitrate. The impregnated samples were dried in an oven at 110 °C for 24 h and then calcined in a muffle furnace at 400 °C for 3 h under a static air atmosphere. The H_2_SO_4_, WO_3_, and ZrO_2_ contents in the modified samples varied between 1 and 15 wt%. The modified samples were named *x*% modifier/K400, where *x* is the weight percentage of the modifier used.

**Table 4:** The chemical composition of K400 catalyst.

(Wt%)	SiO_2_	Al_2_O_3_	Fe_2_O_3_	CaO	MgO	SO_3_	Na_2_O	K_2_O	Mn_2_O_3_	TiO_2_	Loss
K400	44.67	29.87	0.70	7.77	0.38	0.53	0.17	0.22	0.23	0.48	14.98

### Catalyst characterization

Thermal behavior (thermogravimetric (TG) and differential thermal analysis (DTA)) of the raw kaolin clay were studied using a Shimadzu thermal analyzer (Japan, 60H) using air as a heating atmosphere and a heating rate of 10 °C/min.

The structure of the catalysts was identified via X-ray diffraction (XRD) using a Philips diffractometer (model PW 2103/00) equipped with a Ni-filtered Cu Kα radiation (λ = 1.5408 Å). The samples were scanned over the 2*ϴ* range from 4° to 80°, at a scan rate of 2°/min.

Most functional groups of the catalysts were investigated via FTIR in the 4000–400 cm^−1^ region, which recorded on a Nicolet spectrophotometer (model 6700) by using KBr pellet technique. In this technique, the samples were prepared by mixing the catalyst powder with KBr (*w*/w 1:100) together and then compressed into thin pellets. The results were collected at a resolution of 2 cm^−1^.

The catalyst microstructure and crystal morphology analysis were determined using a scanning electron microscope (JEOL Model JSM-5400 LV, Jeol, Tokyo, Japan).

The surface area and texture of the catalysts were determined via N_2_ adsorption at − 196 °C using the gas adsorption apparatus Nova 3200 instrument (Quantachrom Instrument Corporation, USA). The Brunauer–Emmett–Teller (BET) model was used to calculate the surface area.

Isopropyl alcohol (IPA) was dehydrated as previously described by Said et al.^11^, and the pyridine FTIR (Py-FTIR) adsorption technique were used^59^. Those were done to measure the acidity of the catalysts. For pyridine FTIR adsorption experiments, about 30 mg of the samples were grounded and mixed with KBr and then pressed with a pressure in a self-supporting disc in air. The spectra of the samples without pyridine were recorded with a spectral resolution of 2 cm^−1^. Afterward, the discs were heated to 200 °C in a drying oven for 3 h to remove any possible physisorbed species before saturated with pyridine for 7 h after evacuation in desiccator. Then, the pyridine excess was removed for 30 min under vacuum and the spectra were recorded. To determine the bands relevant to Lewis and Brønsted acidic sites, the spectra obtained after pyridine adsorption were subtracted from those obtained before pyridine adsorption (fresh samples)^70^.

### Methanol dehydration

The methanol dehydration reaction was conducted in a conventional fixed-bed flow-type reactor using 500 mg of modified kaolin, 6000 ml/g/h gas hourly space velocity, and 4% methanol in the gas feed. The reaction was conducted in the temperature range of 200–350 °C under atmospheric pressure. The reactant and products were analyzed using a gas chromatograph (Pro-GC Unicam) with a thermal conductivity detector and a 2 m DNP glass column. At least three successive data points were taken for each reaction at equilibrium temperature (1 h). The average of these points was used in calculating the conversion and selectivity values. The methanol conversion and DME selectivity were calculated as previously described^62^.

## Supplementary Information

Supplementary Figure S1.

## Data Availability

All data generated or analyzed during this study are included in this published article.
