# Supplementary material for: Modified natural kaolin clay as an active, selective, and stable catalyst for methanol dehydration to dimethyl ether
Source: Sci Rep. 2022 Jun 7;12:9407. doi: 10.1038/s41598-022-13349-0 (PMC9174221; doi:10.1038/s41598-022-13349-0)
Supplement: Supplementary file 1 — Supplementary Figure S1. [file 41598_2022_13349_MOESM1_ESM.docx]

**Modified natural kaolin clay as an active, selective, and stable catalyst for methanol dehydration to dimethyl ether**

**Mohamed Abd El-Aal^1^, Abd El-Aziz Ahmed said^1*^, Mohamed H. Abdallah^2^, Mohamed Nady Goda^1^**

**^1^Catalysis and Surface Chemistry Lab, Chemistry Department, Faculty of Science, Assiut University, Assiut, 71516 Egypt**

**^2^Chemistry Department, Faculty of Science, Al-Azhar University, Assiut, 71524 Egypt**

*E-mail: [aasaid55@yahoo.com](mailto:aasaid55@yahoo.com), [a.a.said@aun.edu.eg](mailto:a.a.said@aun.edu.eg) (Abd El-Aziz A. Said)

**Fig. S1**. XRD patterns of the fresh and the spent 10% ZrO_2_/K400 catalyst.
